# Supplementary material for: HIVprotI: an integrated web based platform for prediction and design of HIV proteins inhibitors
Source: J Cheminform. 2018 Mar 9;10:12. doi: 10.1186/s13321-018-0266-y (PMC5845081; doi:10.1186/s13321-018-0266-y)
Supplement: Supplementary file 2 — Additional file 2. Source code of HIVProtI web server. [file 13321_2018_266_MOESM2_ESM.zip › HIVprotI_Source-code/hivprotI/weka-3-6-6/doc/weka/core/AttributeStats.html]

AttributeStats


---


|  |  |  |
| --- | --- | --- |
| **Overview** \| **Package** \| **Class** \| **Tree** \| **Deprecated** \| **Index** \| **Help** \| **Weka's home** | |  |
| **PREV CLASS**   **NEXT CLASS** | **FRAMES**    **NO FRAMES**     **All Classes** |  |
| SUMMARY: NESTED \| FIELD \| CONSTR \| METHOD | DETAIL: FIELD \| CONSTR \| METHOD |  |


---


## weka.core Class AttributeStats

```
java.lang.Object
  weka.core.AttributeStats
```

**All Implemented Interfaces:**: java.io.Serializable, RevisionHandler

---

``` public class AttributeStats extends java.lang.Object implements java.io.Serializable, RevisionHandler ```

A Utility class that contains summary information on an
the values that appear in a dataset for a particular attribute.

**Version:**
:   $Revision: 1.10 $

**Author:**
:   Len Trigg

**See Also:**: Serialized Form

---

| **Field Summary** | |
| --- | --- |
| `int` | `distinctCount`             The number of distinct values |
| `int` | `intCount`             The number of int-like values |
| `int` | `missingCount`             The number of missing values |
| `int[]` | `nominalCounts`             Counts of each nominal value |
| `Stats` | `numericStats`             Stats on numeric value distributions |
| `int` | `realCount`             The number of real-like values (i.e. |
| `int` | `totalCount`             The total number of values (i.e. |
| `int` | `uniqueCount`             The number of values that only appear once |


**Constructor Summary**

`AttributeStats()`


| **Method Summary** | |
| --- | --- |
| `java.lang.String` | `getRevision()`             Returns the revision string. |
| `java.lang.String` | `toString()`             Returns a human readable representation of this AttributeStats instance. |

**Methods inherited from class java.lang.Object**

`equals, getClass, hashCode, notify, notifyAll, wait, wait, wait`

**Field Detail**

### intCount

```
public int intCount
```

:   The number of int-like values

---


### realCount

```
public int realCount
```

:   The number of real-like values (i.e. have a fractional part)

---


### missingCount

```
public int missingCount
```

:   The number of missing values

---


### distinctCount

```
public int distinctCount
```

:   The number of distinct values

---


### uniqueCount

```
public int uniqueCount
```

:   The number of values that only appear once

---


### totalCount

```
public int totalCount
```

:   The total number of values (i.e. number of instances)

---


### numericStats

```
public Stats numericStats
```

:   Stats on numeric value distributions

---


### nominalCounts

```
public int[] nominalCounts
```

:   Counts of each nominal value


**Constructor Detail**

### AttributeStats

```
public AttributeStats()
```


**Method Detail**

### toString

```
public java.lang.String toString()
```

:   Returns a human readable representation of this AttributeStats instance.

    :   **Overrides:**: `toString` in class `java.lang.Object`
    :   **Returns:**: a String represtinging these AttributeStats.

---


### getRevision

```
public java.lang.String getRevision()
```

:   Returns the revision string.

    :   **Specified by:**: `getRevision` in interface `RevisionHandler`
    :   **Returns:**: the revision


---


|  |  |  |
| --- | --- | --- |
| **Overview** \| **Package** \| **Class** \| **Tree** \| **Deprecated** \| **Index** \| **Help** \| **Weka's home** | |  |
| **PREV CLASS**   **NEXT CLASS** | **FRAMES**    **NO FRAMES**     **All Classes** |  |
| SUMMARY: NESTED \| FIELD \| CONSTR \| METHOD | DETAIL: FIELD \| CONSTR \| METHOD |  |


---
